# The interleukin-1 receptor antagonist influences interleukin-1 effects in rat and mouse

**DOI:** 10.1155/S0962935192000061

**Published:** 1992

**Authors:** P. Bjrök, K. Ohlsson

**Affiliations:** 1 Department of Surgical Pathophysiology University of Lund, Malmö General Hospital Malmö S-214 01 Sweden

## Abstract

In this work we have focused on the ability of interleukin-1 to induce an acute phase protein response and a degranulation of polymorphonuclear leukocytes in vivo. The capacity of the interleukin-1 receptor antagonist to influence these events was also investigated. It was shown that interleukin-1 induced an acute phase protein response in rats and mice. In rats α_2_-macroglubolin levels were increased in plasma after an interleukin-1 injection whereas α_1_-inhibitor-3 decreased in plasma. In the mice plasma amyloid **P** was increased. The interleukin-1 receptor antagonist blocked the increase of α_2_-macroglobulin and plasma amyloid **P** in a dose dependent way while the effect on the α_1_-inhibitor-3 decrease was less pronounced. Interleukin-1 led to polymorphonuclear leukocyte degranulation in vivo as measured by increased cathepsin **G** plasma levels. The interleukin-1 receptor antagonist could influence the early phase of this degranulation.


Mediators of Inflammation 1, 27–31 (1992)

### The interleukin-1 receptor antagonist influences interleukin-1 effects in rat and mouse

P. Björk[CA] and K. Ohlsson

Department of Surgical Pathophysiology, University of Lund, Malmö General Hospital, S-214 01 Malmö, Sweden

[CA] Corresponding Author

**In this work we have focused on the ability of interleukin-1 to induce an acute phase protein response and a degranulation of polymorphonuclear leukocytes *in vivo*. The capacity of the interleukin-1 receptor antagonist to influence these events was also investigated. It was shown that interleukin-1 induced an acute phase protein response in rats and mice. In rats $\alpha_2$-macroglubolin levels were increased in plasma after an interleukin-1 injection whereas $\alpha_1$-inhibitor-3 decreased in plasma. In the mice plasma amyloid P was increased. The interleukin-1 receptor antagonist blocked the increase of $\alpha_2$-macroglobulin and plasma amyloid P in a dose dependent way while the effect on the $\alpha_1$-inhibitor-3 decrease was less pronounced. Interleukin-1 led to polymorphonuclear leukocyte degranulation *in vivo* as measured by increased cathepsin G plasma levels. The interleukin-1 receptor antagonist could influence the early phase of this degranulation.**

**Key words:** $\alpha_1$-inhibitor-3, $\alpha_2$-macroglobulin, Acute phase response, Amyloid P, Cathepsin G, Interleukin-1, Interleukin-1 receptor antagonist, Polymorphonuclear leukocytes, Proteinase inhibitors, Proteinases

### Introduction

Different forms of tissue injury cause the host to react with a uniform acute phase response.[1] This acute phase response is effected by a variety of mediators including different cytokines. Among the cytokines, interleukin-1 (IL-1), interleukin-6 (IL-6) and tumour necrosis factor $\alpha$ (TNF $\alpha$) have been shown to hold central positions in the acute phase response.[2] Several plasma proteins are increased or decreased as a part of the acute phase response. This acute phase protein response differs from species to species. In humans two proteinase inhibitors, $\alpha_1$-antichymotrypsin and $\alpha_1$-proteinase inhibitor ($\alpha_1$-antitrypsin), are among the strongest reacting acute phase proteins. Their main function is to inhibit polymorphonuclear leukocyte (PMN) cathepsin G and elastase, respectively.[3]

In the rat $\alpha_2$-macroglobulin ($\alpha_2$M) is a marked acute phase protein[4] and in the mouse amyloid P shows highly increased plasma levels in the acute phase response.[5] The purpose of the present paper was to evaluate the effect of the IL-1 receptor antagonist (IL-1ra) on some IL-1 mediated processes *in vivo*.

### Methods

*Rat cathepsin G enzyme-linked immunosorbent assay (ELISA):* Rat PMN cathepsin G was isolated as described[6] and a monospecific polyclonal rabbit anti-rat cathepsin G antiserum was produced as described.[7] An IgG-fraction was prepared on a Protein G Sepharose 4 Fast Flow (Pharmacia LKB Biotechnology, S-751 82 Uppsala, Sweden) according to the manufacturer's instruction. The IgG-fraction was characterized by double diffusion.[8] 1% (w/v) agarose in 0.07 M barbital buffer containing 0.2 M calcium lactate and 1.0 M NaCl was cast to 1 mm thickness and incubated for 2 days in a humid chamber. The gel was washed, dried and stained with Coomassie. The result is given in Fig. 1.

In the ELISA E.I.A. II Plus Microtitration plates (Flow Laboratories, McLean, VA 22102, USA) were used. In the first incubation step 100 $\mu$l of rabbit anti-rat cathepsin G antibody diluted in 0.05 M carbonic buffer with 0.02% (w/v) $NaN_3$, pH 9.8 was incubated per well overnight at 4°C. The plates were washed with phosphate buffered saline, containing 0.05% (v/v) Tween 20, pH 7.4, between each incubation. In the second step 200 $\mu$l 0.01 M sodium phosphate buffer, with 0.5 M NaCl, 0.1% Tween 20, 0.02% (w/v) $NaN_3$ and 2.0% (w/v) bovine serum albumin (BSA), pH 7.4 was incubated at room temperature for 0.5–2 h. In the third step 100 $\mu$l standard and plasma samples were incubated for 2 h at 37°C. Diisopropylfluorophosphate (Fluka Chemie Ag, CH-9470 Buchs, Switzerland) inactivated rat cathepsin G served as standard. Standard samples were 0, 1.56, 3.12, 6.25, 12.5 and 25 $\mu g\,l^{-1}$. Rat plasma was diluted 5, 10 and 20 times routinely. Both standard samples and plasma samples were diluted in 0.01 M sodium phosphate buffer, with 0.5 M NaCl, 0.1% (v/v)

© 1992 Rapid Communications of Oxford Ltd

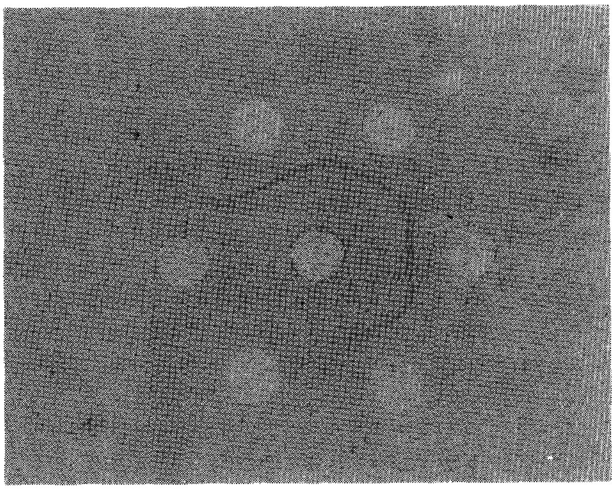

FIG. 1. Double diffusion characterization of rabbit anti-rat cathepsin G antiserum. Samples were: central basin; rat PMN extract; outer basins, four basins contained different rabbit anti-rat cathepsin G IgG-fractions, two basins were empty.

Tween 20, 0.02% (w/v) $NaN_3$ and 1.0% (w/v) BSA, pH 7.4. In the fourth step 100 μl biotinylated[8] IgG fractioned rabbit anti-cathepsin G antibody 10 mg $l^{-1}$ was diluted 1/100 in the same buffer as for samples and incubated for 3 h at 37°C. In the fifth step 100 μl alkaline phosphatase conjugated avidin (Dakopatts, DK-2600, Glostrup, Denmark) was diluted 1/500 in 0.01 M phosphate buffer, with 0.01% (v/v) Tween and 0.02% $NaN_3$, pH 7.4 and incubated at room temperature for 0.5 h. Finally 100 μl substrate solution [3.8 mM *p*-nitrophenyl phosphate (Sigma) in 9.7% (v/v) diethanolamine/HCl buffer containing 0.5 mM $CaCl_2$, pH 9.8] was added and incubated at room temperature. The absorbance at 405 nm was read in a Titertek Multiscan spectrophotometer. Standard curve and sample values were calculated by a Titersoft ELISA personel computer program (Flow Laboratories, Rickmansworth, WD3 1PQ, UK).

*Antisera:* Rabbit anti-rat $\alpha_2$M and rabbit anti-rat $\alpha_1$-inhibitor-3 ($\alpha_1I_3$) were prepared as described[9,10] and rabbit anti-mouse amyloid P was obtained from Synergen Inc. CO 80301, USA. The different proteins were measured by electroimmunoassay.[11]

*Animal experiments:* Male Wistar rats (Møllegaard Avelslaboratorium A/S, DK-4623 Skensved, Denmark) weighing 275–325 g were used. The animals were conscious during the whole experimental time and had free access to water and standard food pellets. Recombinant human IL-1β was prepared by recombinant DNA technology in *E. coli* (Synergen Inc.). In the 48 h experiments 0.5 μg or 5 μg IL-1 in 1 ml phosphate buffered saline was given as a single intraperitoneal (i.p.) injection at 0 h. One group that received 5 μg IL-1 was given a subsequent single i.p injection of 5 mg recombinant human IL-1 receptor antagonist (IL-1ra) in phosphate buffered saline at 0 h. IL-1ra was prepared by recombinant DNA technology in *E. coli* (Synergen Inc.). Blood samples of approximately 0.5 ml were taken from the tail into tubes containing EDTA. Plasma was immediately prepared and frozen at −70°C. Samples were taken; 0, 12, 24, 36 and 48 h.

In the 12 h experiments 5 μg IL-1 in 1 ml phosphate buffered saline was given as a single i.p injection at 0 h. After the IL-1 injection 5 or 25 mg IL-1ra in 1 ml phosphate buffered saline was given as a subsequent single i.p. injection. One group did not receive any IL-1ra. Blood samples of approximately 0.5 ml were taken from the tail into tubes containing EDTA. Plasma was immediately prepared and frozen at −70°C. Samples were taken at 0, 1, 3, 6 and 12 h.

To study plasma amyloid P in mice, Balb/c strain (Møllegaard Avelslaboratorium A/S) was used. Four different groups with five animals in each received 0.5 μg IL-1 in 100 μl phosphate buffered saline i.p. as a single injection. Immediately after the injection of IL-1, 0, 0.5, 5 or 10 mg IL-1ra in 100 μl phosphate buffer was given in a single i.p. injection. At 0 and 20 h blood samples were collected from the tails. EDTA plasma was prepared and frozen at −70°C until analysed. During the experimental time the animals had free access to water and standard food pellets. The animal experiments were sanctioned by the local ethical committee for animal experiments.

*Statistical methods:* Student's *t*-test (paired groups, two tailed) was used to test whether and when groups treated with the IL-1ra were different from corresponding control. $p < 0.05$ was regarded as statistically significant.

### Results

*Rat cathepsin G ELISA:* Precision as inter-assay coefficient of variation for a plasma standard ($n = 16$) and intra-assay coefficient of variation for a plasma standard ($n = 20$) was 10% and 7%, respectively. The sensitivity was 1.5 μg $l^{-1}$.[12] Normal rat plasma concentration was less than 1.5 μg $l^{-1}$. When diisopropylfluorophosphate inactivated cathepsin G was added to normal rat plasma 90% of the added amount could be measured with the assay. Dilution-curves of standard samples and plasma samples with increased levels were parallel.

*Cathepsin G in IL-1 stimulated rats:* The animals did not show any outer signs of discomfort during the whole experimental time. Plasma levels of cathepsin G were increased 1 h after injection of IL-1. Rats injected with 25 mg IL-1ra did not show increased

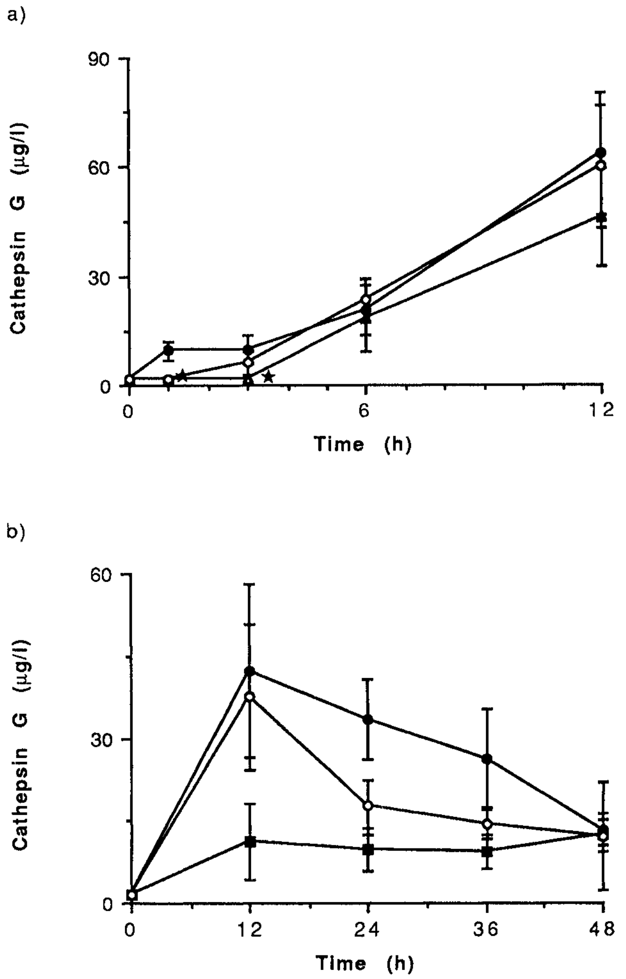

FIG. 2. (a) Plasma cathepsin G in rats stimulated with 5 μg IL-1 in a single i.p. injection at 0 h. One group (●—●) only received IL-1 and two groups also received 5 mg IL-1ra (○—○) and 25 mg IL-1ra (△—△), respectively. IL-1ra was administered as a single injection i.p. at time point 0. Results are given as plasma cathepsin G μg $l^{-1}$ (mean ± SE mean of six rats). * indicates $p < 0.05$ compared to untreated rats. (b) Plasma cathepsin G in rats stimulated with 0.5 μg (■—■) or 5 μg (●—●) IL-1 by an i.p. injection. One group stimulated with 5 μg IL-1 also received 5 mg IL-1ra (○—○) administered by the same route. Results are given as plasma cathepsin G μg $l^{-1}$ (mean ± SE mean of five rats).

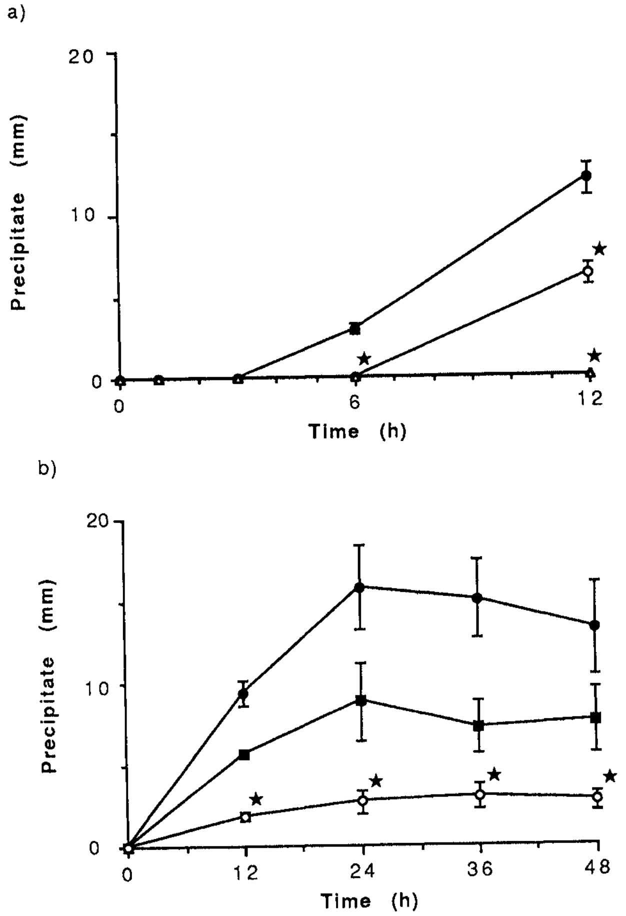

FIG. 3. (a) Plasma level of $\alpha_2$M in rats stimulated with 5 μg (●—●) IL-1 by an i.p. injection. Two groups also received 5 mg IL-1ra (○—○) and 25 mg IL-1ra (△—△), respectively, administered by the same route. Results are given as mm precipitate height in electroimmunoassay (mean ± SE mean of six rats). * indicates $p < 0.05$ compared to untreated rats. (b) Plasma level of $\alpha_2$M in rats stimulated with 0.5 μg (■—■) or 5 μg (●—●) IL-1 by an i.p. injection. One group stimulated with 5 μg IL-1 also received 5 mg IL-1ra (○—○) administered by the same route. Results are given as mm precipitate height in electroimmunoassay (mean ± SE mean of five rats). * indicates $p < 0.05$ compared to untreated rats.

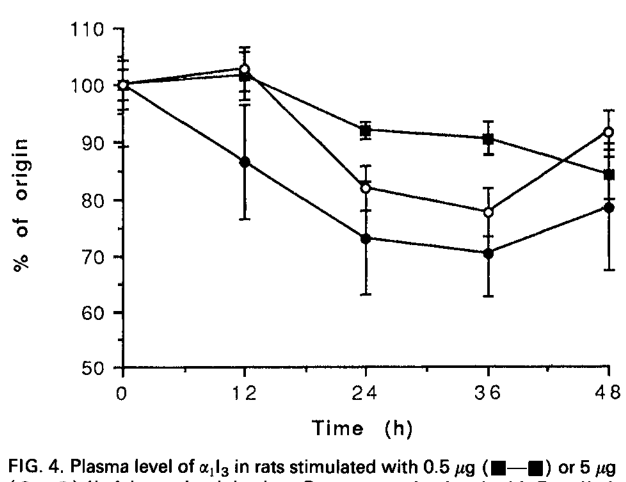

FIG. 4. Plasma level of $\alpha_1I_3$ in rats stimulated with 0.5 μg (■—■) or 5 μg (●—●) IL-1 by an i.p. injection. One group stimulated with 5 μg IL-1 also received 5 mg IL-1ra (○—○) administered by the same route. Results are given as mm precipitate height in electroimmunoassay (mean ± SE mean of five rats).

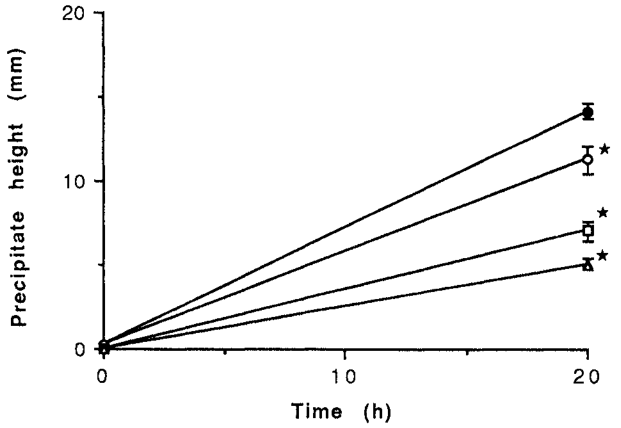

FIG. 5. Plasma level of amyloid P in mice stimulated with 0.5 μg IL-1 administered by an i.p. injection. After the IL-1 injection the four groups received different amounts of IL-1ra; 0 mg IL-1ra (●—●), 0.5 mg IL-1ra (○—○), 5 mg IL-1ra (□—□) and 10 mg IL-1ra (△—△). IL-1ra was given by the same route as IL-1. Results are given as mm precipitate height in electroimmunoassay (mean ± SE mean of five mice). * indicates $p < 0.05$ compared to untreated mice.

plasma levels until 6 h after the injection of IL-1 (Fig. 2a). In rats followed 48 h after the IL-1 injection a peak value was seen after 12 h (Fig. 2b). Hereafter the plasma level declined. Rats treated with IL-1ra reached the same peak level at 12 h but showed lower plasma values at 24 and 36 h compared to untreated rats. These differences were not statistically significant. $\alpha_2$M measured by electroimmunoassay were detectable in plasma from rats at 6 h after injection of IL-1 and reached a maximum level after 24 h. IL-1ra administration caused a dose dependent decrease of this response (Fig. 3a and 3b).

$\alpha_1I_3$ plasma levels decreased in all rats despite administration of IL-1ra but the decrease in plasma levels was less pronounced in rats that received IL-1ra (Fig. 4). The lesser decreased plasma levels seen in IL-1ra treated rats were not statistically significant. Mice stimulated with IL-1 showed an increase in plasma amyloid P levels at 20 h after the stimulation of IL-1. Treatment with IL-1ra could diminish this increase in a dose dependent manner. Results are shown in Fig. 5.

### Discussion

In this study we have focused on the ability of IL-1 to induce an acute phase protein response and degranulation of PMNs *in vivo*. The human IL-1ra potential to influence these events in the rat and mouse was also investigated.

Tissue damage emerging from inflammation, neoplasia, trauma, burn injury and operation trauma induces an acute phase response in the host.[1] This response includes fever, metabolic changes and increase or decrease of certain plasma proteins. Increased protein synthesis during an acute phase response is due to stimulation of the hepatocytes by IL-1, TNF$\alpha$ and IL-6. The latter cytokine is thought to play a key role in the human acute phase protein response.[13] In the rat one of the fastest and strongest reacting acute phase proteins is $\alpha_2$M.[4] Normally plasma level is approximately 50 $\mu$g l$^{-1}$, which is just below the detection limit for an electroimmunoassay. In the acute phase response the plasma levels may increase to 0.5–5 g l$^{-1}$.

After a single injection of IL-1 i.p. a detectable plasma level of $\alpha_2$M was seen after 6 h and at 24 h a maximum level was reached. Thereafter the plasma levels decreased. These increased plasma levels seen are in agreement with plasma levels and increased mRNA hepatocyte levels seen in rats stimulated with turpentine.[4]

Rat $\alpha_1I_3$ is a proteinase inhibitor with a normal serum concentration of 7–9 g l$^{-1}$.[14] In an inflammatory process the serum level is decreased.[10] Transcription activity in the liver follows this decrease in serum level.[4] Thus, $\alpha_1I_3$ is regarded as a negative acute phase reactant. A single IL-1 injection into rats resulted in a decreased plasma level reaching a minimum level after 36 h.

In the mouse an i.p. single injection of IL-1 gave a significant raised plasma level of amyloid P, 20 h after the injection. This is in agreement with a raised plasma level seen after subcutaneous turpentine injection in mice.[5]

IL-1ra[15] is a pure receptor antagonist to IL-1. It has recently been isolated in its native form[16] and also cloned.[17,18] IL-1ra has been shown to influence inflammatory processes in experimental models.[19–21]

When rats received a single i.p. injection subsequent to a prior injection of IL-1 it was possible to totally block the increase of $\alpha_2$M plasma level. This blockade was seen when a 5 000-fold molar excess of IL-1ra was given and lasted for at least 12 h. When lesser amounts of IL-1ra were given the increased $\alpha_2$M plasma level could not be totally blocked. Decreased plasma levels of $\alpha_1I_3$ after stimulation by IL-1 were not altered in IL-1ra treated rats.

In mice a single i.p. injection of IL-1 resulted in increased plasma amyloid P level. Administration of a single dose IL-1ra by the same route diminished this increase of amyloid P in a dose dependent way. When a 20 000-fold excess of IL-1ra over IL-1 was given the increase of plasma level was reduced to 35% of the one obtained without IL-1ra. The data presented so far, clearly demonstrates the ability of IL-1ra to compete with IL-1 in binding to the IL-1 receptor in rat and mouse *in vivo*.

The difference in the capacity of IL-1ra to influence the positive respective the negative acute phase protein response in IL-1 stimulated animals illustrate the complexity of the protein synthesis during the acute phase response. It may suggest an occurrence of receptor subtypes for IL-1 on hepatocytes or on cells capable of producing e.g. IL-6 and TNF.

Human PMNs possess a high affinity receptor for human recombinant IL-1$\alpha$ to the number of approximately 700 per cell.[22] IL-1$\beta$ and rhIL-1ra have also been shown to bind to this type II IL-1 receptor.[23] *In vitro* human PMNs have been shown to degranulate when stimulated by IL-1 purified from monocytes.[24] However, these results could not be confirmed when recombinant IL-1 was used as a stimulating agent.[25] The explanation for the difference in results may be due to the difficulty in obtaining absolutely pure IL-1 preparations from monocyte-cultures. Our results clearly demonstrate that recombinant IL-1 administered i.p. in the rat led to a degranulation from PMNs *in vivo* as measured by increased cathepsin G plasma level. The release of cathepsin G by stimulation of IL-1 was dose related. Cathepsin G is a major enzymatic constituent in PMN,[26] located in the azurophile granulas.[27] The highest plasma level was measured 12 h after the i.p. injection of IL-1. Since IL-1 is capable of inducing production of other inflammatory mediators[28] the fact that IL-1 induces PMN degranulation *in vivo* but not *in vitro* may be due to this mechanism. However, IL-1 may also act directly on the PMNs as a primer for degranulation. Recently recombinant IL-1 has been shown to enhance formyl-methionyl-leucylphenylalanine induced release of myeloperoxidase.[29] Administration of a 5 000-fold excess of IL-1ra over IL-1 blocked the release of cathepsin G for 3 h. After 12 h the highest cathepsin G plasma levels were measured, independently of whether the rats had received IL-1ra or not. These results may be caused by a subsequent mobilization of PMNs from the bone marrow.

Recently it has been shown that both PMN cathepsin G and elastase are capable of degrading TNF$\alpha$ and TNF$\beta$ *in vitro* but not IL-1$\alpha$.[30] IL-1 shows many effects both as a single mediator and also in concert with other cytokines e.g. TNF$\alpha$. The capability of IL-1 to induce degranulation of at least cathepsin G *in vivo* may reflect a regulatory function of PMN proteinases on TNF. In the bloodstream the proteinases are inactivated by proteinase inhibitors but locally in tissues they may be enzymatically active and thus be able to degrade proteins.

### References

1. Kushner I, Ganapathi M, Schultz D. The acute phase response is mediated by heterogeneous mechanisms. *Ann NY Acad Sci* 1989; **557**: 19–30.
2. Dinarello CA. Interleukin-1 and its biologically related cytokines. *Adv Immunol* 1989; **44**: 153–205.
3. Travis J. Structure, function, and control of neurophil proteinases. *Am J Med* 1988; **84 (Suppl 6A)**: 37–42.
4. Schreiber G, Tsykin A, Aldred AR, *et al.* The acute phase response in the rodent. *Ann NY Acad Sci* 1989; **557**: 61–86.
5. Gershenwald JE, Fong Y, Fahey III TJ, *et al.* Interleukin-1 receptor blockade attenuates the host inflammatory response. *Proc Natl Acad Sci USA* 1990; **87**: 4966–4970.
6. Björk P, Ohlsson K. Purification and N-terminal amino-acid sequence analysis of rat polymorphonuclear cathepsin G. *Biol Chem Hoppe-Seyler* 1990; **371**: 595–601.
7. Björk P, Axelsson L, Ohlsson K. Release of dog polymorphonuclear leukocyte cathepsin G, normally and in endotoxin and pancreatitic shock. *Biol Chem Hoppe-Seyler* 1991; **372**: 419–426.
8. Ochterlony Ö. Diffusion-in-gel methods for immunological analysis. *Prog Allergy* 1958; **5**: 1–78.
9. Gauthier F, Mouray H. Rat $\alpha_2$ acute-phase macroglobulin isolation and physicochemical properties. *Biochem J* 1976; **159**: 661–665.
10. Gauthier F, Ohlsson K. Isolation and some properties of a new enzyme-binding protein in rat plasma. *Hoppe-Seyler's Z Physiol Chem* 1978; **359**: 987–992.
11. Laurell C-B. Electroimmuno assay. *Scand J Clin Lab Invest* 1972; **29(Suppl 124)**: 21–37.
12. Rodbard D, Lewald JE. Computer analysis of radioligand assay and radioimmunoassay data. *Acta Endocrinol* 1970; **64(Suppl 147)**: 79–103.
13. Heinrich PC, Castell JV, Andus T. Interleukin-6 and the acute phase response. *Biochem J* 1990; **265**: 621–636.
14. Lonberg-Holm K, Reed DL, Roberts RC, *et al.* Three high molecular weight protease inhibitors of rat plasma. *J Biol Chem* 1987; **262**: 438–445.
15. Dinarello CA. Interleukin-1 and interleukin-1 antagonism. *Blood* 1991; **77**: 1627–1652.
16. Hannum CH, Wilcox CJ, Arend WP, *et al.* Interleukin-1 receptor antagonist activity of a human interleukin-1 inhibitor. *Nature* 1990; **343**: 336–340.
17. Eisenberg SP, Evans RJ, Arend WP, *et al.* Primary structure and functional expression from complementary DNA of a human interleukin-1 receptor antagonist. *Nature* 1990; **343**: 341–346.
18. Carter DB, Deibel Jr MR, Dunn CJ, *et al.* Purification, cloning, expression and biological characterization of an interleukin-1 receptor antagonist protein. *Nature* 1990; **344**: 633–638.
19. Ohlsson K, Björk P, Bergenfeldt M, *et al.* Interleukin-1 receptor antagonist reduces mortality from endotoxin shock. *Nature* 1990; **348**: 550–552.
20. Cominelli F, Nast CC, Clark BD, *et al.* Interleukin-1 (IL-1) gene expression, synthesis and effect of specific IL-1 receptor blockade in rabbit immune complex colitis. *J Clin Invest* 1990; **86**: 972–980.
21. Wakabayashi G, Gelfand JA, Burke JF, *et al.* A specific receptor antagonist for interleukin-1 prevents *Escherichia coli*-induced shock in rabbits. *FASEB J* 1991; **5**: 338–343.
22. Rhyne JA, Mizel SB, Taylor RG, *et al.* Characterization of the human interleukin-1 receptor on human polymorphonuclear leukocytes. *Clin Immunol Immunopathol* 1988; **48**: 354–361.
23. Granowitz EV, Clark BD, Mancilla J, *et al.* Interleukin-1 receptor antagonist competitively inhibits the binding of interleukin-1 to the type II interleukin-1 receptor. *J Biol Chem* 1991; **266**: 14147–14150.
24. Smith RJ, Bowman BJ, Speziale SC. Interleukin-1 stimulates granule exocytosis from human neutrophils. *Int J Immunopharmac* 1986; **8**: 33–40.
25. Georgilis K, Schaefer C, Dinarello CA, *et al.* Human recombinant interleukin 1$\beta$ has no effect on intracellular calcium or on functional responses of human neutrophils. *J Immunol* 1987; **138**: 3403–3407.
26. Bretz U, Baggiolini M. Biochemical and morphological characterization of azurophil and specific granules of human neutrophilic polymorphonuclear leukocytes. *J Cell Biol* 1974; **63**: 251–269.
27. Ohlsson K, Olsson I, Spitznagel K. Localization of chymotrypsin-like cationic protein, collagenase and elastase in azurophil granules of human neutrophilic polymorphonuclear leukocytes. *Hoppe-Seyler's Z Physiol Chem* 1977; **358**: 361–366.
28. Zoja C, Wang JM, Bettoni S, *et al.* Interleukin-1$\beta$ and tumor necrosis factor-$\alpha$ induce gene expression and production of leukocyte chemotactic factors, colony-stimulating factors, and interleukin-6 in human mesangial cells. *Am J Pathol 1991;* **138**: 991–1003.
29. Dularay B, Elson CJ, Clements-Jewery S, *et al.* Recombinant human interleukin-1$\beta$ primes human polymorphonuclear leukocytes for stimulus-induced myeloperoxidase release. *J Leukoc Biol* 1990; **47**: 158–163.
30. Scuderi P, Nez PA, Duerr ML, *et al.* Cathepsin-G and leukocyte elastase inactivate human tumor necrosis factor and lymphotoxin. *Cell Immunol* 1991; **135**: 299–313.

ACKNOWLEDGEMENTS. We wish to thank Iréna Ljungkrantz for her excellent technical assistance and Dr RC Thompson, Synergen Inc., for providing human recombinant interleukin-1$\beta$ and human recombinant interleukin-1 receptor antagonist. This work was supported by research funds from: The Swedish Medical Research Council (3910); The Medical Faculty, University of Lund; The Swedish Medical Society; The Swedish Society for Medical Research.

**Received 15 October 1991;**
**accepted after revision 14 November 1991**
